# Correction: School performance in Danish children exposed to maternal type 1 diabetes in utero: A nationwide retrospective cohort study

**DOI:** 10.1371/journal.pmed.1004021

**Published:** 2022-06-03

**Authors:** Anne Lærke Spangmose, Niels Skipper, Sine Knorr, Tina Wullum Gundersen, Rikke Beck Jensen, Peter Damm, Erik Lykke Mortensen, Anja Pinborg, Jannet Svensson, Tine Dalsgaard Clausen

The tenth author’s name is incorrect. The correct name is: Tine Dalsgaard Clausen.

The tenth author’s affiliations are incorrect. The correct affiliations are: Department of Gynaecology and Obstetrics, Nordsjællands Hospital, Hillerød, Denmark, Department of Clinical Medicine, University of Copenhagen, Denmark.
