# Supplementary figures and images for: School performance in Danish children exposed to maternal type 1 diabetes in utero: A nationwide retrospective cohort study
Source: PLoS Med. 2022 Apr 26;19(4):e1003977. doi: 10.1371/journal.pmed.1003977 (PMC9041831; doi:10.1371/journal.pmed.1003977)

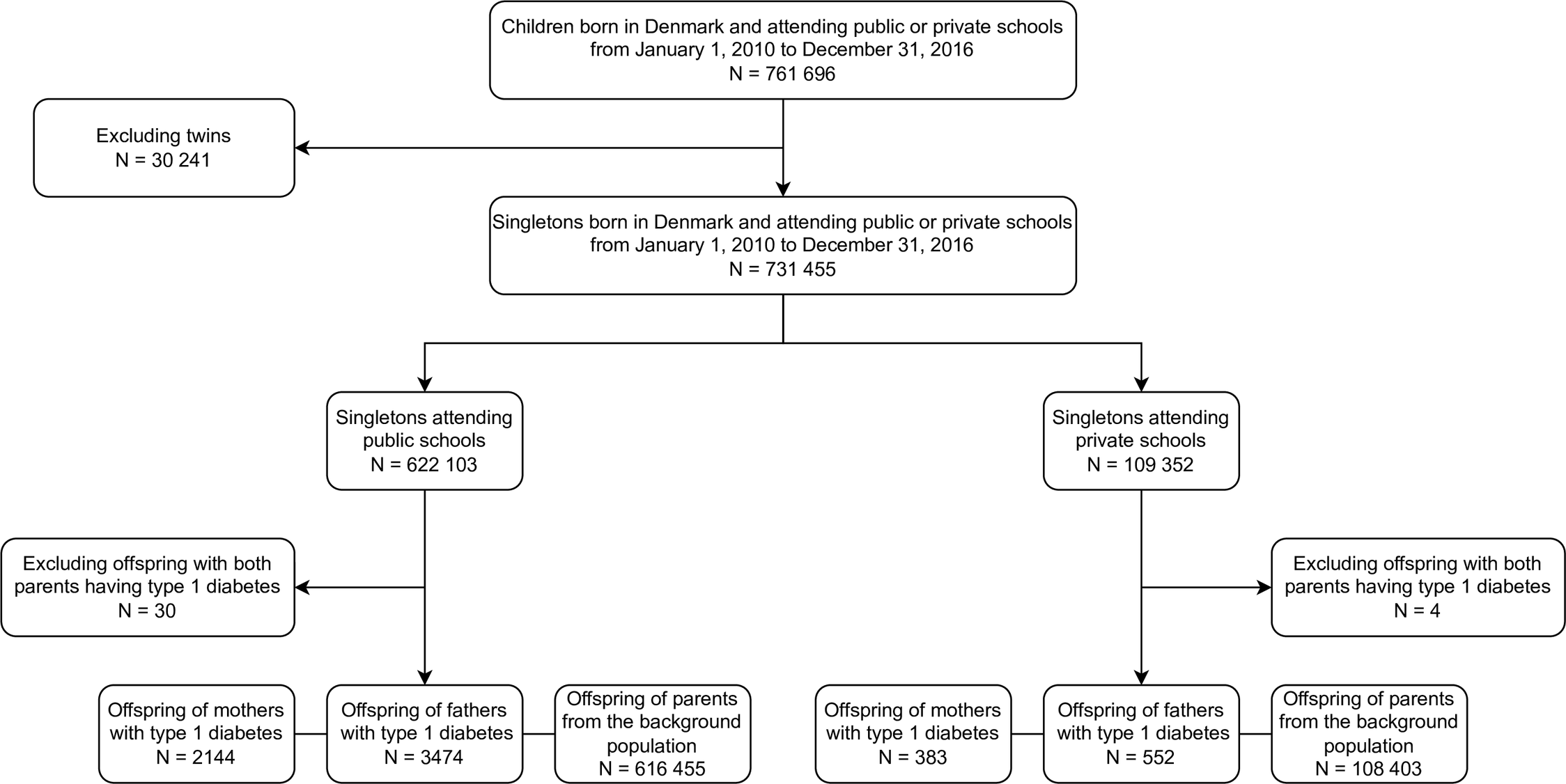

Supplement: S1 Fig — (TIF) [file pmed.1003977.s001.tif]
