# Supplementary material for: School performance in Danish children exposed to maternal type 1 diabetes in utero: A nationwide retrospective cohort study
Source: PLoS Med. 2022 Apr 26;19(4):e1003977. doi: 10.1371/journal.pmed.1003977 (PMC9041831; doi:10.1371/journal.pmed.1003977)
Supplement: S1 Text — (DOCX) [file pmed.1003977.s002.docx]

# *Analysis plan:*

*School performance in Danish children exposed to maternal type 1 diabetes in utero: a nationwide retrospective cohort study*

Date: June 23, 2020

| **Aim:** To investigate the potential adverse effect of intrauterine hyperglycemia on school performance in children born to women with type 1 diabetes taking potential perinatal- and socioeconomic confounders, mediators and effect modifiers into account. |
| --- |
| **Data needed for the study:**   - *Population:* all children attending public schools from 2010–2016 (2, 3, 4, 6, 8^th^ grade)   - *Inclusion criteria:*     - singletons attending school public schools from 2010–2016 (2, 3, 4, 6, 8^th^ grade) - Exposure variable = parental diabetes status at time of delivery   1. children of mothers diagnosed with type 1 diabetes before labor (O-mDM1)   2. children of fathers diagnosed with diabetes before the birth of their children (O-fDM1)   3. children where both parents were diagnosed with type 1 diabetes before delivery (O-mfDM1)   4. children from the background population (O-BP) - *Main outcome:*   - Pooled test scores in math and reading (2, 3, 4, 6, 8^th^ grade) - *Secondary outcomes:*   - test scores in math (3, and 6^th^ grade)   - test scores reading (2, 3, 4, 6, 8^th^ grade)   - trend in test score over the years - *Potential confounding variables*:   - *Parental*:     - maternal and paternal age at delivery     - maternal and paternal SES (educational level, ethnicity, cohabiting parents, income)     - maternal smoking during pregnancy   - *Perinatal covariates*:     - parity     - child sex   - *Other*:     - child type 1 diabetes before time of test - *Potential mediators*: gestational age, LGA/AGA/SGA, mode of delivery (caesarean delivery, vaginal delivery, instrumental delivery), hypertensive disorders of pregnancy, low APGAR score at 5 min, children with major malformations |
| **Plan for analyses (overall strategy):**   - *Comparisons*:   - 1. children of mothers diagnosed with type 1 diabetes before labour   will be compared with:   - - 2. children of fathers diagnosed with diabetes before the birth of their children   - 3. children where both parents were diagnosed with type 1 diabetes before delivery   - 4. children from the background population. - ***Descriptive data****:* Background characteristics will be presented as numbers and percentages, means and standard deviations or medians and interquartile ranges. No p-values on background characteristics will be presented. - ***Main outcome****:* Test scores will be compared in univariate and multiple linear regression analyses. Covariates will be chosen based on current literature on factors influencing school grades.   - Univariate model     - parental diabetes status at time of delivery   - Multiple models     - Model 1 (basic covariates):       - parental diabetes status at time of delivery       - maternal and paternal age       - parity       - child sex       - child type 1 diabetes       - maternal smoking during pregnancy     - Model 2: Model 1 +       - maternal and paternal SES (educational level, ethnicity, cohabiting parents, income)       - hypertensive disorders of pregnancy       - mode of delivery (caesarean delivery, vaginal delivery, instrumental)       - APGAR score at 5 min (low score: < 7)     - Model 3a-b (Potential obstetrics and perinatal mediators): Model 2 + covariate effect + interaction effect of the following covariates with parental diabetes status at time of delivery       - 3a: gestational age (< 224 days, 224–258, 259–279, > 279)       - 3b: birthweight according to gestational age (LGA/AGA/SGA, defined as 90% percentile from the expected sex-specific birth weight for the given gestational age [Marsal et al., 1996])     - Sensitivity analyses on mean test score, Model 2:       - Model 2 excluding children with major malformations - ***Secondary outcomes*:** Test scores will be compared in univariate and multiple linear regression analyses (Model 1 and 2) using linear regression. Covariates will be chosen based on current literature on factors influencing school grades.   - test scores in math (3, and 6^th^ grade)   - reading (2, 3, 4, 6, 8^th^ grade)   - trend in test score over the years |

**Modifications to the original analysis plan for the presented analyses**

The presented analyses are consistent with those planned in the original analysis plan described above, except for the modifications presented below.

- Children where both parents were diagnosed with type 1 diabetes before delivery (O-mfDM1) were excluded from the analyses as this group was too small to study (n=30).
- Reading test scores were analyzed in 2, 4, 6, 8th grade and not 3rd grade as reading is not tested in 3rd grade.
- The trend in test scores over the years was not analyzed as these trends were assumed to be independent of parental diabetes status. Further, relevant changes over time are arguably captured in the analyses by grade.
- Ethnicity was not included in the model as this information is not available in Danish registers. However, immigrant or descendent status was included in our multivariable model, which to some extent counts as a proxy of ethnicity or race.
- Information on instrumental delivery was not included in the covariate “mode of delivery” as several of the co-authors found it would be too detailed information for a potential confounder
- A grade-, topic-, and year-specific fixed effect was added to all adjusted regression models.
- Information on number of siblings was added to Model 1
- Information on maternal and paternal age was moved to Model 2 as we decided that it fitted better together with parental socioeconomic factors.
- Information on hypertensive disorders of pregnancy, mode of delivery, and APGAR score at 5 min was moved to Model 3 as these are considered potential mediators.
- An interaction effect of the potential mediators with parental diabetes status at time of delivery was not performed as co-authors found it too comprehensive and out of scope for the present study**.**
- Sensitivity analyses excluding children with major malformations was not performed as we later decided that this would potentially introduce bias.
- The likelihood of missing a test despite attending a public school during follow-up was assessed together with the likelihood of attending a private school to check if the studied population was representative.
- The models were renamed to optimize the overview.
  - Univariate model was renamed to Model 1
  - Model 1 was renamed Model 2
  - Model 2 was renamed Model 3
  - Model 3a–b was renamed Model S1–S6
- According to the comments of reviewers, a school fixed effect regression model was estimated to accommodate clustering of data at the school level.
