# Supplementary material for: School performance in Danish children exposed to maternal type 1 diabetes in utero: A nationwide retrospective cohort study
Source: PLoS Med. 2022 Apr 26;19(4):e1003977. doi: 10.1371/journal.pmed.1003977 (PMC9041831; doi:10.1371/journal.pmed.1003977)
Supplement: S1 Table — O-BP, offspring of parents from the background population; O-fT1D, offspring of fathers with type 1 diabetes; O-mT1D, offspring of mothers with type 1 diabetes. (DOCX) [file pmed.1003977.s004.docx]

| **S1 Table. Multiple Linear regression analyses comparing test scores in offspring of mothers (O-mT1D) and fathers (O-fT1D) with type 1 diabetes compared with offspring in the background population (O-BP), mean test score difference (95% CI) – Model 3 with sequential adjustment for potential mediators** | | | | | | | | |  |
| --- | --- | --- | --- | --- | --- | --- | --- | --- | --- |
|  |  | | | **Model (n= 1 704 447 test scores)** | | | |  |  |
| Explanatory variables | Model S1 | Model S2 | Model S3 | | | Model S4 | Model S5 | Model S6 | |
|  | Mean diff. (95% CI),  p-value | Mean diff. (95% CI),  p-value | Mean diff. (95% CI),  p-value | | | Mean diff. (95% CI),  p-value | Mean diff. (95% CI),  p-value | Mean diff. (95% CI),  p-value | |
| **Diabetes status** |  |  |  | | |  |  |  | |
| O-mT1D | –1.38(–2.26 to –0.50), p=0.002 | –1.37 (–2.25 to –0.48), p<0.001 | –1.58 (–2.46 to –0.70), p<0.001 | | | –1.30(–2.18 to –0.41), p<0.004 | –2.09 (–2.97 to –1.21), p<0.001 | –1.57 (–2.46 to –0.68),  p=0.001 | |
| O-fT1D | –0.78 (–1.48 to –0.08), p=0.03 | –0.79 (–1.59 to –0.09),  p<0.001 | –0.79 (–1.49 to –0.09),  p=0.03 | | | –0.80 (–1.50 to –0.10),  p=0.03 | –0.80 (–1.50 to –0.10),  p=0.02 | –0.80 (–1.50 to –0.10),  p=0.024 | |
| O-BP | (ref.) | (ref.) | (ref.) | | | (ref.) | (ref.) | (ref.) | |
| **Obstetric and Perinatal Covariates** |  |  | |  |  |  |  |  | |
| Hypertensive disorders of pregnancy (0/1) | –1.46 (–1.72 to –1.20), p<0.001 |  | |  |  |  |  | –1.11 (–1.37 to –0.85),  p<0.001 | |
| Cesarean section (0/1) |  | –0.55 (–0.69 to –0.40),  p<0.001 |  | | |  |  | –0.41 (–0.56 to –0.27),  p<0.001 | |
| Low APGAR score at 5 minutes (<7) (0/1) |  |  | –2.12 (–2.83 to –1.42),  p<0.001 | | |  |  | –1.68 (–2.38 to –0.98),  p<0.001 | |
| Gestational age<32+0 weeks |  |  |  | | | (ref.) |  | (ref.) | |
| Gestational age [32+0; 36+6] weeks |  |  |  | | | 0.41 (–0.90 to 1.73),  p=0.54 |  | –0.21 (–1.53 to 1.11),  p=0.76 | |
| Gestational age [37+0; 39+6] weeks |  |  |  | | | 1.24 (–0.05 to 2.54),  p=0.06 |  | 0.31 (–0.99 to 1.61),  p=0.64 | |
| Gestational age > 40+0 weeks |  |  |  | | | 1.37 (0.08 to 2.67),  p=0.04 |  | 0.45 (–0.84 to 1.75),  p=0.49 | |
| Small for gestational age (0/1) |  |  |  | | |  | (ref.) | (ref.) | |
| Average for gestational age (0/1) |  |  |  | | |  | 2.23 (2.07 to 2.40),  p<0.001 | 2.16 (1.99 to 2.33),  p<0.001 | |
| Large for gestational age (0/1) |  |  |  | | |  | 2.99 (2.77 to 3.21),  p<0.001 | 2.99 (2.77 to 3.20),  P<0.001 | |
| **Test: (*P* value)** |  |  |  | | |  |  |  | |
| O-mT1D = O-fT1D | 0.30 | 0.31 | 0.17 | | | 0.38 | 0.03 | 0.18 | |
| Notes: Differences are adjusted for grade-, topic-, and year specific fixed effects, offspring sex, parity, number of siblings, offspring with type 1 diabetes, maternal smoking during pregnancy, parental highest educational level, income, immigrant or descendant status, age, and parents living together (corresponding to Model 3).  Model S1 is further adjusted for hypertensive disorders of pregnancy.  Model S2 is further adjusted for cesarean section.  Model S3 is further adjusted for low APGAR score at 5 minutes (<7).  Model S4 is further adjusted for gestational age.  Model S5 is further adjusted for birth weight according to expected sex-specific birth weight for the given gestational age.  Model S6 is with simultaneous adjustment for all potential mediators in Model S1–S5.  *P* value from Wald test (F-test) of equality of the regression coefficients to maternal and paternal diabetes is reported. | | | | | | | | |  |
