# Supplementary material for: School performance in Danish children exposed to maternal type 1 diabetes in utero: A nationwide retrospective cohort study
Source: PLoS Med. 2022 Apr 26;19(4):e1003977. doi: 10.1371/journal.pmed.1003977 (PMC9041831; doi:10.1371/journal.pmed.1003977)
Supplement: Attachment — Submitted filename: Response to reviewers.docx [file pmed.1003977.s005.docx]

Dear Editors of PLOS Medicine,

Thank you very much for showing interest and taking your time to comment on our manuscript entitled “School performance in Danish children exposed to maternal type 1 diabetes in utero: a nationwide retrospective cohort study” (manuscript no PMEDICINE-D-21-04566R1). We are grateful for the thorough review and have now considered all the comments.

On behalf of all the co-authors

Sincerely

Anne Lærke Spangmose

-----------------------------------------------------------

Dear Dr. Spangmose,

Thank you very much for submitting your manuscript "School performance in Danish children exposed to maternal type 1 diabetes in utero: a nationwide cohort study" (PMEDICINE-D-21-04566R1) for consideration at PLOS Medicine.

Your paper was evaluated by a senior editor and discussed among all the editors here. It was also discussed with an academic editor with relevant expertise, and sent to independent reviewers, including a statistical reviewer. The reviews are appended at the bottom of this email and any accompanying reviewer attachments can be seen via the link below:

In light of these reviews, I am afraid that we will not be able to accept the manuscript for publication in the journal in its current form, but we would like to consider a revised version that addresses the reviewers' and editors' comments. Obviously we cannot make any decision about publication until we have seen the revised manuscript and your response, and we plan to seek re-review by one or more of the reviewers.

In revising the manuscript for further consideration, your revisions should address the specific points made by each reviewer and the editors. Please also check the guidelines for revised papers at <http://journals.plos.org/plosmedicine/s/revising-your-manuscript> for any that apply to your paper. In your rebuttal letter you should indicate your response to the reviewers' and editors' comments, the changes you have made in the manuscript, and include either an excerpt of the revised text or the location (eg: page and line number) where each change can be found. Please submit a clean version of the paper as the main article file; a version with changes marked should be uploaded as a marked up manuscript.

In addition, we request that you upload any figures associated with your paper as individual TIF or EPS files with 300dpi resolution at resubmission; please read our figure guidelines for more information on our requirements: <http://journals.plos.org/plosmedicine/s/figures>. While revising your submission, please upload your figure files to the PACE digital diagnostic tool, <https://pacev2.apexcovantage.com/>. PACE helps ensure that figures meet PLOS requirements. To use PACE, you must first register as a user. Then, login and navigate to the UPLOAD tab, where you will find detailed instructions on how to use the tool. If you encounter any issues or have any questions when using PACE, please email us at [PLOSMedicine@plos.org](mailto:PLOSMedicine@plos.org).

We expect to receive your revised manuscript by Feb 10 2022 11:59PM. Please email us (plosmedicine@plos.org) if you have any questions or concerns.

***Please note while forming your response, if your article is accepted, you may have the opportunity to make the peer review history publicly available. The record will include editor decision letters (with reviews) and your responses to reviewer comments. If eligible, we will contact you to opt in or out.***

We ask every co-author listed on the manuscript to fill in a contributing author statement, making sure to declare all competing interests. If any of the co-authors have not filled in the statement, we will remind them to do so when the paper is revised. If all statements are not completed in a timely fashion this could hold up the re-review process. If new competing interests are declared later in the revision process, this may also hold up the submission. Should there be a problem getting one of your co-authors to fill in a statement we will be in contact.  YOU MUST NOT ADD OR REMOVE AUTHORS UNLESS YOU HAVE ALERTED THE EDITOR HANDLING THE MANUSCRIPT TO THE CHANGE AND THEY SPECIFICALLY HAVE AGREED TO IT. You can see our competing interests policy here: <http://journals.plos.org/plosmedicine/s/competing-interests>.

Please use the following link to submit the revised manuscript:

<https://www.editorialmanager.com/pmedicine/>

Your article can be found in the "Submissions Needing Revision" folder.

To enhance the reproducibility of your results, we recommend that you deposit your laboratory protocols in protocols.io, where a protocol can be assigned its own identifier [1] such that it can be cited independently in the future. Additionally, PLOS ONE offers an option to publish peer-reviewed clinical study protocols. Read more information on sharing protocols at <https://plos.org/protocols?utm_medium=editorial-email&utm_source=authorletters&utm_campaign=protocols>

Please ensure that the paper adheres to the PLOS Data Availability Policy (see <http://journals.plos.org/plosmedicine/s/data-availability)>, which requires that all data underlying the study's findings be provided in a repository or as Supporting Information. For data residing with a third party, authors are required to provide instructions with contact information for obtaining the data. PLOS journals do not allow statements supported by "data not shown" or "unpublished results." For such statements, authors must provide supporting data or cite public sources that include it.

We look forward to receiving your revised manuscript.

Sincerely,

Beryne Odeny,

PLOS Medicine

plosmedicine.org

-----------------------------------------------------------

Requests from the editors:

ANSWER:

A great thanks to the editor for your thorough review of our manuscript and valuable comments. In the following, we have responded to all the comments and revised the manuscript accordingly.

1) Please revise your title according to PLOS Medicine's style. Please consider “School performance in Danish children exposed to maternal type 1 diabetes in utero: a nationwide retrospective cohort study”

ANSWER:

We have changed the title according to PLOS Medicine’s style.

Line 1–3: “School performance in Danish children exposed to maternal type 1 diabetes in utero: a nationwide retrospective cohort study”

2) The Data Availability Statement (DAS) requires revision. For each data source used in your study, if the data are not freely available, please describe briefly the ethical, legal, or contractual restriction that prevents you from sharing it. Please also include an appropriate contact (web or email address) for inquiries (this cannot be a study author).

ANSWER:

Data Availability Statement

The data are drawn from a restricted access data source and cannot be made publicly available. The data used in the paper are administrative Danish data maintained by Statistics Denmark, which is kept in a secure server. However, the data can be accessed remotely from within Danish universities and research institutions. If a researcher at a university or other research institution outside Denmark wishes to use these data, this may be accomplished by visiting a Danish research institution or cooperating with researchers or research assistants working in Denmark. For inquiries, visit [www.dst.dk](http://www.dst.dk) or e-mail: dst@dst.dk.

3) Please move the ethical approval statement from the end of the main text to the Methods section.

ANSWER:

The ethical approval statement has been moved to the Method section.

Line 220–222: “*Ethical approval*

The study was approved by the Danish Data Protection Agency. This approval constitutes the necessary legal requirement, and informed consent is not required.”

4) Abstract:

a) Please combine the Methods and Findings sections into one section, “Methods and findings”

ANSWER:

The two sections have been combined.

Line 48–59: “Methods and findings: Population-based retrospective cohort study from 2010 to 2016. Nationally standardised school test scores (range, 1–100) were obtained for public school grades 2, 3, 4, 6, and 8 in O-mT1D and compared with those in O-fT1D and O-BP. Of the 622 073 included children, 2144 were O-mT1D and 3474 O-fT1D. Multiple linear regression models were used to compare outcomes, including the covariates offspring with type 1 diabetes, parity, number of siblings, offspring sex, smoking during pregnancy, parental age, and socioeconomic factors. Mean test scores were 54.2 (SD 24.8) in O-mT1D, 54.4 (SD 24.8) in O-fT1D, and 56.4 (SD 24.7) in O-BP. In adjusted analyses, the mean differences in test scores were −1.59 (95% CI −2.48 to −0.71, p<0.001) between O-mT1D and O-BP, and −0.78 (95% CI −1.48 to −0.08, p=0.03) between O-fT1D and O-BP. No significant difference in the adjusted mean test scores was found between O-mT1D and O-fT1D (p=0.16).

The study’s limitation was no access to measures of glycaemic control during pregnancy.”

b) Please ensure that all numbers presented in the abstract are present and identical to numbers presented in the main manuscript text.

ANSWER:

This has been done. Please note that some numbers have been updated after re-estimating regression models using a school fixed effect regression model.

c) Please quantify the main results (with 95% CIs and p values).

ANSWER:

All main results have been quantified.

d) In the last sentence of the Abstract Methods and Findings section, please describe the main limitation(s) of the study's methodology.

ANSWER:

The main limitation of the study has been added to the abstract.

Line 59: “The study’s limitation was no access to measures of glycaemic control during pregnancy.”

e) Conclusions: Please address the study implications without overreaching what can be concluded from the data and emphasize what is new considering the current evidence.

ANSWER:

The conclusion has been rephrased:

Line 60–61: “O-mT1D achieved lower test scores than O-BP but similar test scores compared with O-fT1D.”

f) Please remove the funding statement from the end of the abstract

ANSWER:

The funding statement has been removed.

5) At this stage, we ask that you include a short, non-technical Author Summary of your research to make findings accessible to a wide audience that includes both scientists and non-scientists. The Author Summary should immediately follow the Abstract in your revised manuscript. This text is subject to editorial change and should be distinct from the scientific abstract. Please see our author guidelines for more information: <https://journals.plos.org/plosmedicine/s/revising-your-manuscript#loc-author-summary>

ANSWER:

An Author Summary has been added to the manuscript.

Line 63–91:

## “Author Summary

### Why was this study done?

- The influence of maternal diabetes during pregnancy on offspring cognition has been widely explored because high blood sugar levels in pregnant women are suspected to affect fetal development, including the brain.
- Limited evidence is available on how different subtypes of maternal diabetes are associated with offspring cognition (eg, gestational diabetes and type 1 and type 2 diabetes).
- Limited data are available on the association between maternal type 1 diabetes during pregnancy and offspring cognition using offspring of fathers with type 1 diabetes as the unexposed reference group, which allows for adjustments of potentially shared genes and familial stress of having a parent suffering from a serious chronic disease like diabetes.

### What did the researchers do and find?

- Data were identified from Danish registers, and test scores in math (grades 3 and 6) and reading (grades 2, 4, 6, and 8) were obtained on all Danish children attending public schools from 2010 to 2016.
- We included 2144 offspring of mothers and 3474 offspring of fathers with type 1 diabetes, and 616 455 from the background population, including 1 704 447 test scores.
- Offspring of mothers with type 1 diabetes achieved lower test scores than offspring in the background population but similar test scores compared with offspring of fathers with type 1 diabetes.

### What Do These Findings Mean?

- The lower test scores in the offspring of mothers with type 1 diabetes appear to reflect a negative association of having a parent with type 1 diabetes rather than a specific adverse effect of maternal type 1 diabetes during pregnancy on the fetus.
- This study presents evidence of an alternative explanation for the previously observed adverse effect of maternal type 1 diabetes during pregnancy on offspring cognitive development.
- These results are reassuring for women with type 1 diabetes, as one of their main concerns is whether dysregulation of diabetes during their pregnancy may cause impaired cognitive development in their coming children.”

6) Please avoid assertions of primacy ("… to provide among/ some of the first evidence...."). Instead, state “to our knowledge” or similar.

ANSWER:

The manuscript has been revised accordingly.

7) Please conclude the Introduction with a clear description of the study hypothesis.

ANSWER:

The hypothesis has been added to the Introduction section.

Line 113–114:

“We hypothesised that O-mT1D would achieve lower test scores compared with O-fT1D and O-BP due to intrauterine exposure of hyperglycaemia.”

8) Did your study have a prospective protocol or analysis plan? Please state this (either way) early in the Methods section.

ANSWER:

Our analysis plan has been added as a supplementary text (Text S1). A statement has been made in the method section. Furthermore, a legend has been included at the end of the manuscript.

Line 117–118:

“The analysis plan was drafted prospectively in June 2020 (Text S1).”

Line 396–397:

“**Text S1**: Prospective analysis plan: School performance in Danish children exposed to maternal type 1 diabetes in utero: a nationwide retrospective cohort study.”

a) If a prospective analysis plan (from your funding proposal, IRB or other ethics committee submission, study protocol, or other planning document written before analyzing the data) was used in designing the study, please include the relevant prospectively written document with your revised manuscript as a Supporting Information file to be published alongside your study, and cite it in the Methods section. A legend for this file should be included at the end of your manuscript.

ANSWER:

Please see our answer to question 8.

b) If no such document exists, please make sure that the Methods section transparently describes when analyses were planned, and when/why any data-driven changes to analyses took place.

ANSWER:

Please see our answer to question 8.

c) In either case, changes in the analysis-- including those made in response to peer review comments-- should be identified as such in the Methods section of the paper, with rationale.

9) Please state whether the health data were de-identified (anonymized) prior to access.

ANSWER:

A sentence has been added to the Method section:

Line 128–129:

“All data were de-identified prior to access.”

10) Please ensure that the study is reported according to the STROBE guideline, and include the completed STROBE checklist as Supporting Information. Please add the following statement, or similar, to the Methods: "This study is reported as per the Strengthening the Reporting of Observational Studies in Epidemiology (STROBE) guideline (S1 Checklist)."
The STROBE guideline can be found here: <http://www.equator-network.org/reporting-guidelines/strobe/>

ANSWER:

The STROBE guideline has been added as a supplementary checklist (Checklist S1). A statement has been made in the method section. Furthermore, a legend has been included at the end of the manuscript.

Line 118–120:

“This study is reported as per the Strengthening the Reporting of Observational Studies in Epidemiology (STROBE) guideline (Checklist S1).”

Line 398–399:

“**Checklist S1:** Strengthening the Reporting of Observational Studies in Epidemiology (STROBE) guideline.”

11) When completing the STROBE checklist, please use section and paragraph numbers, rather than page numbers.

12) Please avoid assertions of primacy (“This is the first study to...."). Instead, state “to our knowledge” or similar.

ANSWER:

The manuscript has been revised accordingly.

13) Was ethnicity or race considered in this study?

ANSWER:

Ethnicity or race was not considered in our study as this information is not available in Danish registers. However, immigrant or descendent status was included in our multivariable model, which to some extent counts as a proxy of ethnicity or race.

14) In your statistical analyses, please account for data clustering at the school level. Generalized Estimating Equations (GEE) or hierarchical/ multilevel models, among others, may be useful in this case.

ANSWER:

All regression models were re-estimated using a school fixed effect regression model, to account for clustering of data at the school level. In practice, this was done using the ‘areg’ command in STATA absorbing the school indicator variable. We think that the fixed effect model is a sensible choice to model the unobserved time-invariant heterogeneity on the level of the schools. As seen from the updated set of results, changing to this model only had a modest impact. We now specify in the methods section that we estimate a regression model with school fixed effects.

A sentence has been added to the method section.

Line 187–189:

“To accommodate clustering of data at the school level, a school fixed effect regression model was estimated.”

15) We note the potential for unobservable confounding in this observational study. Have you considered using robust methods such as propensity score matching to address this?

ANSWER:

We decided to implement ordinary least squares (OLS) regression for a couple of reasons. First of all, we do not think that we would credibly account for all the residual, unobserved confounders by doing, e.g., propensity score matching. With this in mind, using OLS seemed like the simplest modelling choice. Note that all the covariates in our regression models are binary variables. In this way, we are in principle controlling for the covariates non-parametrically, avoiding making assumptions about the covariates entering the model linearly. We also think that the primary role of adding covariates (i.e., going from Model 1–3) is to minimize residual variance (by adding variables that explain school outcomes) to gain precision in the estimates. Looking at our results in retrospect, none of our conclusions changed depending on we went from having no covariates to the full set of covariates. The estimates on maternal and paternal diabetes do not change dramatically. In this light, we do expect that propensity score matching would produce qualitatively or quantitatively different results.

16) Please discuss whether missing scores were missing (at random/ completely at random) and the justification for your approach to handling missing data.

ANSWER:

Test scores were not missing at random. For example, school children with a lot of absenteeism would be more likely to miss a test. A priori, we would expect missing a test to be associated with poor socioeconomic status (through higher absenteeism for instance). But we did not know before conducting the study if there would be an association between parental diabetes and missing a test. The first sensitivity check assigns counterfactual test scores when these are missing that are as low as possible. Hence, we are assuming that those who miss a test are those who perform worst of all. If children with missing test scores are overrepresented with either type of parental diabetes status, we would get an idea of maximum difference in test scores we could obtain had they taken the test. The second sensitivity check reweights the estimates by upweighting the covariates that are predictive of missing a test.

17) In the results text and tables, please quantify the main results with both 95% CIs and p values (where applicable).

ANSWER:

P values have been added to the main text and the tables.

18) Throughout the text, please remove language that implies causality, such as “more likely explained by” in the discussion and conclusion sections. Refer to associations instead.

ANSWER:

The manuscript has been revised accordingly.

19) References:
a) Please select the PLOS Medicine reference style in your citation manager. In-text reference call outs should be presented as follows noting the absence of spaces within the square brackets: "... countries [1,2]."
b) In the reference section, please ensure six names appear before et al.

ANSWER:

The references (in text and reference section) have been changed according to PLOS Medicine reference style.

20) Please remove the “Data sharing”, “Declaration of Interests”, “transparency”, and “Funding” statements at the end of the main text. This information is captured in the metadata obtained in the submission form.

ANSWER:

The paragraphs have been removed.

Comments from the reviewers:

Dear Reviewer #1,

Thank you for your thorough review of our manuscript and valuable comments.

Reviewer #1: "School performance in Danish children exposed to maternal type 1 diabetes in utero: a nationwide cohort study" studies both the possible adverse effects of mothers with Type 1 diabetes (O-mT1D, n=2,144) and fathers with Type 1 diabetes (O-fT1D, n=3,474) on their offspring, against a background population (O-BP, n=616,455). This was performed as a population-based retrospective cohort study with data from 2010 to 2016 using nationally-standardized test scores for reading and math, at various public school grade levels. Adjusted (pooled) analyses found statistically-significant (adverse) mean test score difference of -1.62 for o-mT1D and −0.86 for O-fT1D, against the control group. Various sensitivity analyses involving different sets of covariates were attempted (Table 2, Supplement Table 1), with generally consistent outcomes for the T1D vs. control groups.

The scale of the study and the examination of fathers with Type 1 diabetes are particular strengths, with the latter relatively novel to the area, and relevant to answering hypotheses about potential mechanisms for the observed adverse effects relating to non-maternal factors. Some issues might however be considered:

1. Inclusion/exclusion and group criteria might be considered to be summarized in a flowchart if possible.

ANSWER:

A flowchart has been added as a supplementary figure (Supplementary fig. 1). Furthermore, a legend has been included.

2. Related to the above, the treatment of other diabetes types (Type 2, gestational, etc.) in both parents and offspring might be clarified, prefably also in the flowchart if included. Were they excluded/covariates?

ANSWER:

Thank you for this relevant question. Parents or offspring with type 2 diabetes or mothers with gestational diabetes are not excluded from the analyses. This could potentially cause an *underestimation* of the negative association between maternal type 1 diabetes during pregnancy and offspring test scores in the comparisons between O-mT1D and O-BP as offspring of mothers with type 2 diabetes or gestational diabetes are included in the background population. In the comparison of O-mT1D and O-fT1D, some mothers of O-fT1D would have been diagnosed with either type 2 diabetes or gestational diabetes; hence the negative association between maternal type 1 diabetes during pregnancy and offspring test scores could be *underestimated*. However, we expect the effect of parents with either type 2 diabetes or gestational diabetes in O-BP or O-fT1D to be deluded by the large number of healthy parents in these groups, mainly because very few parents (most importantly mothers) will have developed type 2 diabetes before birth due to their young age. The prevalence of gestational diabetes has increased from 1.7% of the total births in 2004 to 2.9% of the total births in 2012 [2].

Conceptually, we also think that the relevant comparison here is the ‘average child’ in the background population. If we start excluding children where the mother (or father) had other diagnoses than type 1 diabetes, we make the comparison group healthier.

To illustrate that this is not a big issue, in either case, we identified 7,019 mothers who had a confirmed GDM diagnosis prior to the birth of the child. The children of these mothers carried out 23,302 tests. If we exclude these tests from the data and re-estimate the model (Model 3), we get the following results; see Table R1.

| Table R1: Full Model 3 with and without GDM cases | | |
| --- | --- | --- |
| Explanatory variables | GDM included | GDM excluded |
|  | Mean Difference (95% CI), p-value | Mean Difference (95% CI), p-value |
| Diabetes status |  |  |
| O-mT1D | -1.59 (-2.48 to -0.71), p<0.001 | -1.61 (-2.50 to -0.73), p<0.001 |
| O-fT1D | -0.78 (-1.48 to -0.08), p=0.03 | -0.83 (-1.54 to -0.13), p<0.001 |
| O-BP | (ref.) | (ref.) |
| N= | 1,704,447 | 1,681,145 |

As can be seen, excluding these tests had only neglectable impacts on the estimates. No differences were found when comparing O-mT1D and O-fT1D.

Identifying individuals with type 2 diabetes is difficult in the patient registry as patients are often treated by general practitioners [3].

Finally, it is problematic to add information on parental type 2 diabetes or gestational diabetes to the multiple regression analyses or exclude them as both diagnoses are suggested to be associated with socioeconomic factors [4-8]. Furthermore, it is problematic as mothers with type 1 diabetes cannot also be diagnosed with gestational diabetes or type 2 diabetes.

3. In Line 99, it is stated that "Offspring in whom both the mother and the father were diagnosed with diabetes (n=30, Line 192) were excluded". It might be clarified if this refers to T1D only for both, and the number of such cases.

ANSWER:

It refers to only type 1 diabetes. The sentence has been reformulated.

Line 138–140:

“Offspring in whom both the mother and the father were diagnosed with type 1 diabetes were excluded.”

4. In Line 144, it is stated that test scores were analyzed in a pooled analysis, with each test score (assumed for different topics/grades) as a unique observation. It might be discussed as to whether pooling across topics/time is appropriate, for example since reading scores would seem to be represented twice as much as math scores, simply by (arbitrary) virtue of having twice as many tests taken. More details on how the pooling was setup might be provided, possibly in supplementary material. Nonetheless, individual tests appear to generally support the main findings (Figure 1).

ANSWER:

Test scores were pooled independent of topic or grade, hence more reading test scores are included in the pooled analysis. However, as test scores are also analysed individually according to topic or grade, we do not think it is a problem.

5. While some prior work has been cited as justification for the choice of covariates (Line 152), certain common covariates (e.g. age at each test, ethnicity, individual school performance) often considered in studies involving student test scores, appear not to have been included. It might be briefly clarified as to whether such effects are assumed to have been covered by other included covariates.

ANSWER:

We believe that the mentioned covariates are already proxied by the current conditioning set. Age at test is to a large extent captured by the grade level effects; ethnicity is proxied by immigration background (and we do not observe ethnicity directly in the data). As to individual school performance, we are not sure it would be methodologically sound to add that as a covariate, as school performance is the outcome we are exploring (proxied by the test score).

6. In Line 207, it is stated that "The mean of test scores in both math and reading were 54.2 (SD 24.8) in O-mT1D, 54.4 (SD 24.8) in O-fT1D, and 56.4 (SD 24.7) in O-BP". It is assumed that the reported figures are the means for (math and reading) together, and not that the individual means (and SD) were identical; as such, the phrasing might be considered to be reworded (including whether the weighing for math and reading were identical), and results for individual topics might be considered for discussion.

ANSWER:

The reported figures are the means for both math and reading together. The sentence has been rephrased.

Line 255–259:

“The mean of test scores were 54.2 (SD 24.8) in O-mT1D, 54.4 (SD 24.8) in O-fT1D, and 56.4 (SD 24.7) in O-BP, corresponding to a mean difference of −1.90 [95% CI −2.87 to −0.94, p<0.001] (Cohen’s d=–0.08) for O-mT1D versus O-BP, and −1.57 [95% CI −2.33 to −0.81, p<0.001] (Cohen’s d=−0.06) for O-fT1D versus O-BP after adjusting for the grade-topic-year-specific effects (table 2, Model 1).”

As stated in the answer to question 4, test scores were pooled independent of topic or grade, and no reweighting has been done. Hence more reading test scores are included in the pooled analysis.

In the method section, the pooling of test scores has been described (Line 184–187): “As a child could take multiple tests, hence, contributing with multiple test scores (math or reading, but also at different grade levels), test scores were analysed in a pooled analysis, with each test score as a unique observation and cluster-robust standard errors were used to adjust for within-individual correlations.” We can add a light description to the result section on request.

Minor issues:

7. In Line 88, "inclusion of only offspring born in Denmark in whom there was..." might be considered to be reworded to avoid the phrase "only offspring", which might suggest children without siblings, if that is not the intended meaning.

ANSWER:

The sentence has been rewritten.

Line 123–125:

“Offspring were initially identified through the national Medical Birth Register to ensure inclusion of offspring born in Denmark in whom there was valid information on maternal and paternal diabetes diagnosed before pregnancy.”

Reviewer #2: School performance in Danish children exposed to maternal type 1 diabetes in utero: a nationwide cohort study

Overall:

An interesting twist to examination of the effect of in utero exposure to type 1 diabetes is described by using a comparison group of fathers with type 1 diabetes rather than just those offspring without exposure to type 1 diabetes in utero. The linking of databases in Denmark provides a unique opportunity to examine the outcomes of interest.
Children with both parents having type 1 diabetes were excluded, n=30.

Dear Reviewer #2,

Thank you for your thorough review of our manuscript and valuable comments.

From what I can tell rates of diabetes were increasing in the female population of reproductive age during the gestation of the offspring studied, mostly type 1 but there could have been an increase in type 2 and gestational diabetes. My question is, were these offspring, from women with type 2 or gestational diabetes during the pregnancy, removed from the comparison groups?

ANSWER:

The issue of different types of diabetes was also raised by reviewer #1, question 2. Please see the answer to this question.

The introduction brings up the point that there are few human studies that examine the type 1, type 2 and gestational diabetes separately and these are only small studies. I would have thought the authors may have been able to distinguish between the type of in utero diabetes exposure in this population using ICD codes. These fetus' are exposed to hyper- and hypo-glycemia also. I feel that these offspring do not belong in the comparison groups. However, given the sample size this may be a moot point, but at least worth mentioning in the limitations.

ANSWER:

The issue of different types of diabetes was also raised by reviewer #1, question 2. Please see the answer to this question.

Specific questions:

Lines 116-117: I am not sure of the importance of the likelihood of attending a private school as an outcome. I see the use of the likelihood of missing a test in the public school but am not clear about the former. Reporting of the results lines181 to 183 clarifies this. Please make it clear in the methods the reasoning for this "outcome", although I believe it is more a matter of checking that the population under study is representative.

ANSWER:

Thank you for this comment. Some children in Denmark attend private schools, for example, due to special needs. Therefore, we were interested in investigating whether the proportion of children attending private schools differed between the three groups and that no selection has occurred. A sentence has been added to the method section.

Line 157–158:

“Finally, the likelihood of attending a private school was assessed to check if the studied population was representative.”

Lines 190-192: It would be useful to the reader to repeat the number of offspring.
"2144 offspring of 1597 unique mothers and 3474 offspring of 2419 fathers with type 1 diabetes, and 616 455 offspring of 401 406 mothers and 402 785 fathers from the background population were included in the cohort."
Of note - I don't believe they were all adolescents unless I am misunderstanding the population. Mean age at first test was 10.1, but as many had more than one test, I am not sure what age is representative of the population. It might be useful to put age at first test for each group in table 1 under the "Test" as age at test is included in the models.

ANSWER:

Thank you for the suggestion. You are right. Not all of the population is adolescents. The paragraph has been rephrased.

Line 237–239:

“In total, 2144 offspring of 1597 unique mothers and 3474 offspring of 2419 unique fathers with type 1 diabetes, and 616 455 offspring of 401 406 unique mothers and 402 785 unique fathers from the background population had offspring included in the cohort.”

We agree that it can be difficult to describe the average age given that the children/adolescents are tested (potentially) multiple times. Hence, we decided to report the mean age when taking the first test. We decided against reporting more information on age as the tests are already grade specific. From Figure 1 the number of children in each grade can be found. As the school starting age in Denmark is comparable to most other countries, knowing the grade level indicates the average age of the attending children. With this in mind, Figure 1 gives a good indication of the age composition in the sample (beyond a simple metric like the mean age at different test times).

I am surprised by inclusion of both maternal and paternal education and income in model 3, I am presuming collinearity was checked. Sorry, I am confused, in re-reading the notes I see that parental highest education level and income were included in the model. Is that highest income or total, is it a category of percentile rank or a continuous variable? Please clarify. Maybe it would be preferable to only show in the table the variables included in the model.

ANSWER:

We did check for collinearity of the variables. Please also note the relatively narrow confidence bands of the variables related to income and education for both parents. This is an indication that collinearity is not a problem. Please also note that our main exposure variables are not expected to be highly correlated with the (potentially) collinear variables.

In Model 3, we include the highest completed education of the father *and* the mother – i.e., educational status for both of the parents. This is the same with income. The income percentile rank is just an ordering of each mother/father in the income distribution (according to percentile). We then adjust the model by which quarter of the distribution they are placed.

The differences between math and reading results are fascinating and I feel that something is lost by putting both in the same model, there also appear to be grade differences. Model 3 is also used for these analyses, were other models considered?

ANSWER:

The issue of pooling test scores was also raised by reviewer #1. Test scores were pooled independent of topic or grade, hence more reading test scores are included in the pooled analysis. However, as test scores are also analysed individually according to topic or grade, we do not think it is a problem. The analyses were made including covariates from Model 3 as this is the full adjusted model.

Reviewer #3: 
Thank you for the opportunity to review this paper.
Utilising national population data, the authors primarily examined the association between maternal type I diabetes on offspring school performance, but also, in respect to fathers with type1 diabetes and the background population. This is an excellent study and the paper is well written and succinct. Inclusion of fathers with diabetes is particularly important and adds significantly to our understanding of developmental origins of human capital.

ANSWER:

Dear Reviewer #3,

Thank you for your thorough review of our manuscript and valuable comments.

Minor points for consideration:

1. The inclusion of both parity and number of siblings may over-adjust the model - have the authors examined collinearity effects and is there strong enough justification to include both variables?

ANSWER:

The issue of collinearity was also raised by reviewer #2 (although between income and education). In line with the reply to reviewer #2, we investigated for collinearity and noted that collinearity would not bias the coefficients for the main exposure. Note that the confidence bands for number of siblings and parity are very narrow. If they were highly collinear, there would be variance inflation and large confidence bands.

2. As anticipated, sex does have an association with school performance and is appropriate to adjust for. However, there are data suggestion a sexual dimorphism in the effect of maternal glucose on fatal growth, particularly for males. Given the sufficient power of the sample size, it would useful to sex-stratify and examine the association of maternal and paternal diabetes on the math outcome in sex-specific models to test if diabetes exposure effects male school performance more.

ANSWER:

In response to the reviewer’s comment, we estimated a model with an indicator for male interacted with maternal diabetes status for the math test scores (equivalent to estimating sex-specific models). The interaction term was highly insignificant, suggesting that males exposed to maternal type 1 diabetes did not perform differently than girls exposed to maternal type 1 diabetes (similar results were found for reading). We did not add this analysis to the manuscript.

3. Figure 1 is useful to include but the view quality of the figure needs to be improved

ANSWER:

Figure 1 has been improved (layout and quality).

A potential confounder is how well maternal glucose was managed during pregnancy - it is unfortunate that no data could be presented on this, but the authors have discussed this within the limitation section.

References

1.	Laut K, Shepherd L, Radoi R, Karpov I, Parczewski M, Mussini C, et al. Persistent disparities in antiretroviral treatment (ART) coverage and virological suppression across Europe, 2004 to 2015. Euro Surveill. 2018;23(21).

2.	Jeppesen C, Maindal HT, Kristensen JK, Ovesen PG, Witte DR. National study of the prevalence of gestational diabetes mellitus among Danish women from 2004 to 2012. Scand J Public Health. 2017;45(8):811-7.

3.	Schmidt M, Schmidt SA, Sandegaard JL, Ehrenstein V, Pedersen L, Sørensen HT. The Danish National Patient Registry: a review of content, data quality, and research potential. Clin Epidemiol. 2015;7:449-90.

4.	Agardh E, Allebeck P, Hallqvist J, Moradi T, Sidorchuk A. Type 2 diabetes incidence and socio-economic position: a systematic review and meta-analysis. Int J Epidemiol. 2011;40(3):804-18.

5.	Tatulashvili S, Fagherazzi G, Dow C, Cohen R, Fosse S, Bihan H. Socioeconomic inequalities and type 2 diabetes complications: A systematic review. Diabetes Metab. 2020;46(2):89-99.

6.	Kragelund Nielsen K, Damm P, Kapur A, Balaji V, Balaji MS, Seshiah V, et al. Risk Factors for Hyperglycaemia in Pregnancy in Tamil Nadu, India. PLoS One. 2016;11(3):e0151311.

7.	Rönö K, Masalin S, Kautiainen H, Gissler M, Eriksson JG, Laine MK. The impact of educational attainment on the occurrence of gestational diabetes mellitus in two successive pregnancies of Finnish primiparous women: a population-based cohort study. Acta Diabetol. 2020;57(9):1035-42.

8.	Collier A, Abraham EC, Armstrong J, Godwin J, Monteath K, Lindsay R. Reported prevalence of gestational diabetes in Scotland: The relationship with obesity, age, socioeconomic status, smoking and macrosomia, and how many are we missing? J Diabetes Investig. 2017;8(2):161-7.
