# Supplementary material for: School performance in Danish children exposed to maternal type 1 diabetes in utero: A nationwide retrospective cohort study
Source: PLoS Med. 2022 Apr 26;19(4):e1003977. doi: 10.1371/journal.pmed.1003977 (PMC9041831; doi:10.1371/journal.pmed.1003977)
Supplement: Attachment — Submitted filename: Response to reviewers vol. 2.docx [file pmed.1003977.s006.docx]

Dear Editors of PLOS Medicine,

Thank you very much for accepting our manuscript entitled “School performance in Danish children exposed to maternal type 1 diabetes in utero: a nationwide retrospective cohort study” (manuscript no PMEDICINE-D-21-04566R1). We are grateful for the thorough review and have now considered all the comments.

On behalf of all the co-authors

Sincerely

Anne Lærke Spangmose

-----------------------------------------------------------

Dear Dr. Spangmose,

Thank you very much for re-submitting your manuscript "School performance in Danish children exposed to maternal type 1 diabetes in utero: a nationwide retrospective cohort study" (PMEDICINE-D-21-04566R2) for review by PLOS Medicine.

I have discussed the paper with my colleagues and the academic editor and it was also seen again by one reviewer. I am pleased to say that provided the remaining editorial and production issues are dealt with we are planning to accept the paper for publication in the journal.

Please address the remaining issues within 1 week to enable publication of your manuscript in April. The remaining issues that need to be addressed are listed at the end of this email. Any accompanying reviewer attachments can be seen via the link below. Please take these into account before resubmitting your manuscript:

***Please note while forming your response, if your article is accepted, you may have the opportunity to make the peer review history publicly available. The record will include editor decision letters (with reviews) and your responses to reviewer comments. If eligible, we will contact you to opt in or out.***

In revising the manuscript for further consideration here, please ensure you address the specific points made by each reviewer and the editors. In your rebuttal letter you should indicate your response to the reviewers' and editors' comments and the changes you have made in the manuscript. Please submit a clean version of the paper as the main article file. A version with changes marked must also be uploaded as a marked up manuscript file.

Please also check the guidelines for revised papers at <http://journals.plos.org/plosmedicine/s/revising-your-manuscript>for any that apply to your paper. If you haven't already, we ask that you provide a short, non-technical Author Summary of your research to make findings accessible to a wide audience that includes both scientists and non-scientists. The Author Summary should immediately follow the Abstract in your revised manuscript. This text is subject to editorial change and should be distinct from the scientific abstract.

We expect to receive your revised manuscript within 1 week. Please email us ([plosmedicine@plos.org](mailto:plosmedicine@plos.org)) if you have any questions or concerns.

We ask every co-author listed on the manuscript to fill in a contributing author statement. If any of the co-authors have not filled in the statement, we will remind them to do so when the paper is revised. If all statements are not completed in a timely fashion this could hold up the re-review process. Should there be a problem getting one of your co-authors to fill in a statement we will be in contact. YOU MUST NOT ADD OR REMOVE AUTHORS UNLESS YOU HAVE ALERTED THE EDITOR HANDLING THE MANUSCRIPT TO THE CHANGE AND THEY SPECIFICALLY HAVE AGREED TO IT.

Please ensure that the paper adheres to the PLOS Data Availability Policy (see <http://journals.plos.org/plosmedicine/s/data-availability>), which requires that all data underlying the study's findings be provided in a repository or as Supporting Information. For data residing with a third party, authors are required to provide instructions with contact information for obtaining the data. PLOS journals do not allow statements supported by "data not shown" or "unpublished results." For such statements, authors must provide supporting data or cite public sources that include it.

To enhance the reproducibility of your results, we recommend that you deposit your laboratory protocols in [protocols.io](http://protocols.io), where a protocol can be assigned its own identifier (DOI) such that it can be cited independently in the future. Additionally, PLOS ONE offers an option to publish peer-reviewed clinical study protocols. Read more information on sharing protocols at <https://plos.org/protocols?utm_medium=editorial-email&utm_source=authorletters&utm_campaign=protocols>

Please review your reference list to ensure that it is complete and correct. If you have cited papers that have been retracted, please include the rationale for doing so in the manuscript text, or remove these references and replace them with relevant current references. Any changes to the reference list should be mentioned in the rebuttal letter that accompanies your revised manuscript.

Please note, when your manuscript is accepted, an uncorrected proof of your manuscript will be published online ahead of the final version, unless you've already opted out via the online submission form. If, for any reason, you do not want an earlier version of your manuscript published online or are unsure if you have already indicated as such, please let the journal staff know immediately at [plosmedicine@plos.org](mailto:plosmedicine@plos.org).

If you have any questions in the meantime, please contact me or the journal staff on [plosmedicine@plos.org](mailto:plosmedicine@plos.org).

We look forward to receiving the revised manuscript by Mar 23 2022 11:59PM.

Sincerely,

Beryne Odeny, 
PLOS Medicine
[plosmedicine.org](http://plosmedicine.org)

-----------------------------------
1) Abstract conclusion: please add a sentence addressing the study implications for policy and practice.

ANSWER:

A sentence has been added to the abstract.

Line 62–65: “Glycaemic control during pregnancy is essential to prevent various adverse pregnancy outcomes in women with type 1 diabetes. However, the present study reduces previous concerns regarding adverse effects of in-utero hyperglycaemia on offspring cognitive function.”

2) Thank you for providing your STROBE checklist. Please replace the page numbers with paragraph numbers per section (e.g. "Methods, paragraph 1"), since the page numbers of the final published paper may be different from the page numbers in the current manuscript.

ANSWER:

The STROBE checklist has been corrected.

3) In table 2, please define what the income percentile ranks mean e.g. Q1 is which income range etc

ANSWER:

We now specify in table 2 that the Q1 etc. refers to the quartile in the income distribution that the mother or father belongs to. Note that these quartiles are year specific and relate to the given year that the income was measured for the mother/father. Hence, it is difficult to provide any specific income ranges.

4) To help us extend the reach of your research, please provide any Twitter handle(s) that would be appropriate to tag, including your own, your coauthors’, your institution, funder, or lab.

ANSWER:

@Rigshospitalet

@Annelrke

@skipper_niels

@nordsjaelhosp

-------------------------
Requests from Editors:

Comments from Reviewers:

Reviewer #1: We thank the authors for addressing our previous comments. A few further points might be considered:

1. The clarifications for other diabetes types (involving Table R1 in the response) might be included in supplementary material, and/or briefly mentioned in the text, if possible.

ANSWER:

A paragraph has been added:

Line 378–384: "Parents or offspring with type 2 diabetes or mothers with gestational diabetes were not excluded from the analyses. However, we expect the potential effect of parents with either type 2 diabetes or gestational diabetes in O-BP or O-fT1D to be deluded by the large number of healthy parents in these groups, mainly because very few parents will have developed type 2 diabetes before birth due to their young age. Conceptually, we also think that the relevant comparison group is the ‘average child’ in the background population, and by excluding children where the parents had other diagnoses than type 1 diabetes, the comparison group would be made healthier."

2. The test score pooling setup might be described in greater detail, if possible (and offered).

ANSWER:

We believe that the pooling of test scores is described sufficiently in the method section.

3. Limitations relating to available covariates might be briefly discussed.

ANSWER:

A sentence has been added to the discussion section.

Line 364–376: “Ethnicity or race was not considered in our study as this information is not available in Danish registers. However, immigrant or descendent status was included in our multivariable model, which to some extent counts as a proxy of ethnicity or race.”

4. Results as presented in Table 2 appear changed from the previous revision, despite apparently involving the same population. This might be clarified.

ANSWER:

All regression models were re-estimated using a school fixed effect regression model, to account for clustering of data at the school level. This was implemented upon request from the editor.

Any attachments provided with reviews can be seen via the following link:
__________________________________________________
In compliance with data protection regulations, you may request that we remove your personal registration details at any time. (Use the following URL: <https://www.editorialmanager.com/pmedicine/login.asp?a=r>). Please contact the publication office if you have any questions.
